# Treatment Patterns and Health Care Costs in Commercially Insured Patients with Follicular Lymphoma

**DOI:** 10.36469/jheor.2020.16784

**Published:** 2020-09-04

**Authors:** Nathan H. Fowler, Guifang Chen, Stephen Lim, Stephanie Manson, Qiufei Ma, Frank (Yunfeng) Li

**Affiliations:** 1 The University of Texas MD Anderson Cancer Center, Houston, TX, USA; 2 KMK Consulting, Inc., Morristown, NJ, USA; 3 Novartis Pharmaceuticals Corporation, East Hanover, NJ, USA

**Keywords:** treatment patterns, health care costs, follicular lymphoma, treatment Lines, relapsed refractory disease, combination therapy, rituximab monotherapy

## Abstract

**Background:**

Few studies have estimated the real-world economic burden such as all-cause and follicular lymphoma (FL)-related costs and health care resource utilization (HCRU) in patients with FL.

**Objectives:**

This study evaluated outcomes in patients who were newly initiated with FL indicated regimens by line of therapy with real-world data.

**Methods:**

A retrospective study was conducted among patients with FL from MarketScan^®^ databases between January 1, 2010 and December 31, 2013. Patients were selected if they were ≥18 years old when initiated on a FL indicated therapy, had at least 1 FL-related diagnosis, ≥1 FL commonly prescribed systemic anti-cancer therapy after diagnosis, and did not use any FL indicated regimen in the 24 months prior to the first agent. These patients were followed up at least 48 months and the outcomes, including the distribution of regimens by line of therapy, the treatment duration by line of therapy, all-cause and FL-related costs, and HCRU by line of therapy were evaluated.

**Results:**

This study identified 598 patients who initiated FL indicated treatment. The average follow-up time was approximately 5.7 years. Of these patients, 50.2% (n=300) were female, with a mean age of 60.7 years (SD=13.1 years) when initiating their treatment with FL indicated regimens. Overall, 598 (100%) patients received first-line therapy, 180 (43.6%) received second-line therapy, 51 received third-line therapy, 21 received fourth-line therapy, and 10 received fifth-line therapy. Duration of treatment by each line of therapy was 370 days, 392 days, 162 days, 148 days, and 88 days, respectively. The most common first-line regimens received by patients were rituximab (n=201, 33.6%), R-CHOP (combination of rituximab, cyclophosphamide, doxorubicin hydrochloride [hydroxydaunomycin]; n=143, 24.0%), BR (combination of bendamustine and rituximab; n=143, 24.0%), and R-CVP (combination of rituximab, cyclophosphamide, vincristine, and prednisone; n=71, 11.9%). The most common second-line treatment regimens were (N=180): rituximab (n=78, 43.3%) and BR (n=41, 22.8%). Annualized all-cause health care costs per patient ranged from US$97 141 (SD: US$144 730) for first-line to US$424 758 (SD: US$715 028) for fifth-line therapy.

**Conclusions:**

The primary regimens used across treatment lines conform to those recommended by the National Comprehensive Cancer Network clinical practice guidelines. The economic burden for patients with FL is high and grows with subsequent lines of therapy.

## INTRODUCTION

Follicular lymphoma (FL) is an indolent, chronic, slow-growing form of non-Hodgkin’s lymphoma (NHL) and the second most common NHL subtype in the Western hemisphere, accounting for 20% to 25% of NHL cases.^1^–^4^ In the United States, FL has an estimated incidence of 3 to 4 per 100 000 people and accounts for approximately 35% of NHLs.^1^,^5^,^6^ In the United States, the estimated number of new cases in 2016 was 13 960.^5^ Recent evidence indicates that about 30% to 50% of patients will relapse within 5 years of initial diagnosis.^7^,^8^ The incidence varies by ethnicity, with Caucasians being affected more than twice as frequently as other populations.^1^,^9^ The median age at diagnosis is approximately 65 years.^10^

FL is a treatable, yet incurable disease.^11^–^13^ When FL reappears after a period of remission, it is said to relapse.^14^ When the disease does not respond to treatment or the response to treatment is short-lived, it is said to be refractory.^14^ Patients typically receive repeated treatments throughout their lifetime.^12^,^15^ Prior to the initiation of novel therapy, patients may receive several additional lines of treatment.^12^,^16^,^17^ Many treatment choices are listed in the National Comprehensive Cancer Network (NCCN) Clinical Practice Guidelines^®^ in Oncology, but are rarely curative.^12^ Due to the clinical heterogeneity and molecular and morphological diversity of the disease, treatments across lines of therapy tend to be heterogeneous.^12^,^18^ Despite the long remission and disease-free periods that can be achieved with treatment, the disease ultimately relapses in most patients.^19^ In addition, patients who relapse tend to experience shorter response durations to subsequent lines of therapy until they eventually become refractory to treatment.^20^

Currently, there is no consensus on the standard of care for FL patients after second-line therapy.^12^,^21^ At present, rituximab or second-generation anti-CD20 antibodies either as a single agent, as maintenance, or in combination with chemotherapy or stem cell transplant are some of the most effective treatment options for relapsed patients.^4^ Treatment options for patients with FL who relapse after rituximab-containing regimens are limited.^22^,^23^ The aforementioned treatments are expensive. A recent study showed that the annual costs for patients with FL who have progressive disease were about 3.5-fold higher than those with nonprogressive disease.^6^ Furthermore, patients with advanced-stage disease often have higher mortality rates.^6^ For example, a study by Mounier et al. found that patients with stage I/II FL had a 21% mortality rate compared with 28% for patients with stage III/IV disease.^24^ Beyond the financial and clinical burdens of FL,^6^ patients with active relapsed disease tend to have poorer scores than their newly diagnosed counterparts in quality of life (QoL), anxiety, depression, and work productivity.^6^,^25^,^26^ Due to the lack of curative options and the significant financial and QoL burden associated with relapsed disease, new treatment strategies for FL are needed.

Few studies detailing the health care resource utilization (HCRU) and costs associated with lines of therapy for FL are available.^20^,^27^ Most studies have compared the cost-effectiveness of rituximab and first-line therapy or subsequent therapy for patients with FL who have relapsed or refractory disease.^20^,^28^–^41^ Previously, Meyer et al. reported limited data on patients with FL identified in the MarketScan databases in a conference abstract.^20^ Although the patients were grouped together as indolent NHL (which encompassed FL, small lymphocytic lymphoma, marginal zone lymphoma, and lymphoplasmacytic lymphoma), the authors reported that these patients incurred substantial medical costs (US$170 179 in 2013).^20^ A study by Morrison et al. in newly diagnosed patients with FL evaluated data through three lines of therapies for up to 2 years of follow-up,^27^ and reported high HCRU and estimated costs of US$10 460/month. Hence, because extensive HCRU and cost data for third-, fourth-, and fifth-line therapy are currently lacking, this retrospective administrative claims database study evaluated treatment patterns after FL diagnosis and in patients with relapsed and refractory status as well as the economic burden and HCRU and annual health care costs associated with this illness over a 4-year follow-up period.

## METHODS

### Study Design

This retrospective real-world evidence study analyzed MarketScan^®^ Commercial Claims and Encounter and the Medicare Supplemental and Coordination of Benefits databases produced by IBM Watson Health. These secondary databases are fully compliant with the Health Insurance Portability and Accountability Act of 1996.^42^,^43^ This study was designed, implemented, and reported in accordance with the Guidelines for Good Pharmacoepidemiology Practices of the International Society for Pharmacoepidemiology (2016),^44^ the Strengthening the Reporting of Observational Studies in Epidemiology guidelines,^45^ and with the ethical principles contained in the Declaration of Helsinki.^46^ No identifiable protected health information was used in this study.

The MarketScan databases contain deidentified records from millions of patients including Medicare, Medicaid, large employers, managed care organization, hospital, and electronic medical records providers in the United States.^43^ Available data include insurance enrollment status, prescription eligibility information, hospitalization, outpatient visit, physician office visit, prescription drug claims, and related costs. These databases provide detailed diagnosis, treatment, cost (payment), and health care utilization information for health care services performed in inpatient and outpatient settings. Standard demographic variables included age, sex, geographic location, health insurance plan, Charlson Comorbidity Index (CCI) score, and CCI by category.^43^

### Study Population and Selection Criteria

**Figure 1:**
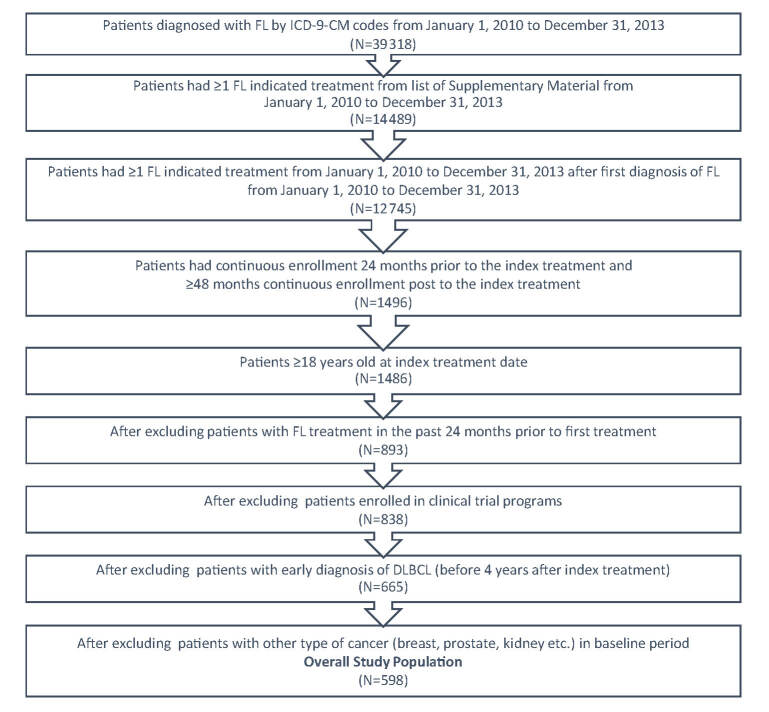
Patient Selection Abbreviations: CM, clinical modification; FL, follicular lymphoma; ICD, International Classification of Diseases.

FL patients were identified within a 3-year period from January 1, 2010 to December 31, 2013 by the International Classification of Diseases (ICD)-9-Clinical Modification-(CM) code 202.0 either as primary or secondary diagnosis from the medical claim database (Figure 1). A list of systemic anti-cancer therapies (Supplementary Material) based on NCCN guidelines was applied to identify patients who received treatment. The first systemic anti-cancer therapy after FL diagnosis was designated as the index therapy and the fill date was designated as index date. To be qualified as newly treated patients, the patients should not have had any FL indicated treatment in the 24 months prior to the index date. The patients were followed for at least 4 years from index date, ending at the earliest of diffuse large B-cell lymphoma (DLBCL) diagnosis, or at the end of continuous enrollment, or until December 31, 2018; the time at which the most recent data were available.

In order to be included in this study, patients must must have satisfied the following criteria: (a) had a diagnosis of at least one FL ICD-9-CM code; (b) had at least one FL indicated treatment; (c) had continuous enrollment 24 months prior to the index treatment, had ≥48 months continuous enrollment post to the index treatment; and (d) were 18 years of age or older at the index treatment date. Patients were excluded if they had one of the following issues: (a) an FL indicated medication utilization in the past 24 months prior to the index treatment; (b) enrollment in clinical trial programs as identified by ICD-9-CM code V70.7 from the data; (c) a diagnosis of DLBCL in the baseline period or in the first four years during the follow-up period; or (d) a diagnosis of malignant neoplasm of the breast, colon, kidney, or prostate in the baseline period.

Since some patients were treated with combination therapies, both monotherapy and combination therapy were identified in the follow-up period. Specifically, the combination therapies were defined if a patient were initiated with more than one agent within one week. Patients were on the same line of therapy if they were treated with the same regimens, which were either mono- or a combination of multiple regimens. Patients were on a different therapy if they added a new therapeutic agent, dropped an agent from a combination therapy, or switched to another agent after 3 months of treatment on current therapies. The duration of therapy was defined as the length of time in days from the beginning of a therapy to the time when the patient switched to another therapy, added another therapy or dropped an agent from current combination therapies, or discontinued their current therapy for more than 90 days except combination therapies in which rituximab was a regimen. The patients were still considered to be on the same therapies if patients continued rituximab as maintenance therapy after dropping other regimens in current rituximab involved combination therapies.

### Study Outcomes

Study outcomes included the distribution of regimens by line of therapy first, second, third, fourth, and fifth line of therapy; treatment duration by line of therapy; and all-cause and FL-related health care costs, including all-cause and FL-related costs by treatment setting. These costs included hospitalization, emergency room (ER) visits, physician office visits, other outpatient visits (including chemotherapy drugs, pathology/lab diagnosis, medicine service, surgery, other miscellaneous outpatient costs) and pharmacy costs. All-cause and FL-related HCRU included all-cause and FL-related hospitalization, all-cause and FL-related length of stay (LOS), all-cause and FL-related ER visits, all-cause and FL-related office visits, and all-cause and FL-related other outpatient visits. All-cause health care costs included medical and pharmacy costs incurred in a particular time frame (eg, first-line costs included costs incurred in the time frame when the patients started their first-line treatment to the day before switching to second-line if patients switched or to the end of follow-up period if patients didn’t switch to next line of treatment). FL-related costs included the medical costs associated with a FL diagnosis in any position and pharmacy costs included costs of treatment listed in the Supplementary Material. All-cause and FL-related HCRU were defined in similar ways. All of these outcomes were analyzed in total and by line of therapy in the follow-up period from the index date to the end of the follow-up period. Since the follow-up period varied for patients in this study, the health care costs were annualized to adjust this variation. Health care costs were measured from a payer’s perspective and were adjusted by consumer price index in 2018 US dollars.

### Statistical Analyses

Only descriptive analyses were conducted for this study. The following statistics were computed: the number and percent of patients on each therapy and by line of therapy, the duration of treatment by each line of therapy, and treatment sequencing. Both all-cause and FL-related health care costs during the follow-up period and by line of therapy included the mean costs, standard deviation (SD) of the costs, median and interquartile range (IQR) of total costs, and by sources of costs (inpatient, ER visits, office visits, and outpatient visits [chemotherapy in outpatient setting, lab/diagnosis, medical services, surgery, other miscellaneous outpatient costs] as well as pharmacy). All-cause and FL-related HCRU computations included the mean, SD of the frequency of hospitalization, the number of days of LOS, the number of ER visits, the number of office visits, and the number of other outpatient visits. Due to the small sample sizes for the fourth- and fifth-lines of treatment, these data were pooled.

## RESULTS

### Patient Attrition and Population Description

A total of 39 318 patients with FL were identified using the ICD-9-CM codes for FL from January 1, 2010 to December 31, 2013 (Figure 1). Of these, 14 489 had ≥1 FL indicated treatment from the list in the Supplementary Material. A total of 598 patients with FL met the study’s inclusion criteria. The mean follow-up time was 5.7 years.

### Patient Baseline Characteristics

In these 598 patients, the mean age of the cohort at initial treatment (index date) was 60.7 years. About half (50.2%) of patients were female and the largest proportion was from the Southern US (36.5%) (Table 1). Most patients (86.1%) had fee-for-service (FFS) health insurance plans. The mean (SD) CCI was 3.2 (1.9) and the most common Charlson comorbidities were other malignancies (95.5%), diabetes without chronic complications (19.4%), and chronic obstructive pulmonary disease (17.1%).

### Treatment Durations

The mean durations of therapy were 370 days (median=141 days) for first-line, 392 days (median=259 days) for second-line, 162 days (median=85 days) for third-line, 148 days (median=42 days) for fourth-line, and 88 (median=21 days) for fifth-line therapies, respectively, (Table 2). Treatment durations for second-line through fifth-line therapies tended to be shorter with each subsequent line of therapy. These treatment durations did not include treatment holidays.

### Distribution of Regimen by Line of Therapy

Since 598 FL-treatment-naïve patients initiated FL indicated therapy, these initial therapies were considered as first-line therapy, of these patients, 180 or 30.1% of initially treated patients received second-line therapy, 51 or 8.5% of initially treated patients received third-line therapy, and 21 or 3.5% of initially treated patients received forth line therapy and 11 or 1.8% initially treated patients received fifth line therapy (Table 3). The most frequently used regimens for first-line therapy were rituximab (33.6%), R-CHOP (combination of rituximab, cyclophosphamide, doxorubicin hydrochloride [hydroxydaunomycin], vincristine sulfate [oncovin] and prednisone, 24.0%), BR (combination of bendamustine and rituximab, 24.0%), and R-CVP (combination of rituximab, cyclophosphamide, vincristine, and prednisone, 11.9%). The most frequently used treatments for second-line therapy were rituximab (43.3%), BR (22.8%), combination of cyclophosphamide and rituximab (7.2%), R-CVP (6.7%), and R-CHOP (6.1%). The most frequently used treatments for third-line therapy were rituximab (31.4%), BR (17.8%), and combination of cyclophosphamide and rituximab (11.8%). There were a few patients on fourth-line and fifth-line therapies counted by each regimen (Table 3).

### All-Cause and FL-Related Health Care Costs

Both all-cause and FL-related health care costs were estimated for all patients. These costs were annualized to adjust the difference in length of follow-up period for patients. The results showed increases in second-line and later therapies (Table 4). In particular, costs were high for third- and fifth-line therapies. A large portion of these increased costs were due to chemotherapy drugs. The all-cause mean of total costs ranged from US$97 141 for first-line, US$125 586 for second-line, US$239 216 for third-line, US$370 597 for fourth-line, and US$424 758 for fifth-line therapies. Chemotherapy drug costs generally made up the highest proportion of medical costs for therapy, accounting for US$55 298 for first-line, US$68 377 for second-line, US$92 648 for third-line, US$163 864 for fourth-line, and US$112 146 for fifth-line therapies.

For FL-related costs, the components that contributed the most to the overall costs were FL-related drugs in any medical setting and FL-related oral medications. FL-related drugs in any medical setting accounted for US$19 990 for first-line, US$19 521 for second-line, US$36 131 for third-line, US$195 616 for fourth-line, and US$14 720 for fifth-line therapies. FL-related oral medications accounted for US$941 for first-line, US$3767 for second-line, US$9586 for third-line, US$85 977 for fourth-line, and US$69 177 for fifth-line therapies.

### Annual Health Care Resource Utilization

HCRU tended to increase in second-line therapies and beyond compared with first-line therapy (Table 5). The number of patients who experienced all-cause hospitalization were 228 (38.1%) for first-line, 56 (31.1%) for second-line, 19 (37.3%) for third-line, 10 (47.6%) for fourth-line, and 5 (50%) for fifth-line therapy, respectively. The annualized mean number of all-cause hospitalizations for these patients in corresponding line of therapy was 0.2 (SD=1.0), 0.5 (2.6), 0.5 (1.1), 0.7 (1.4), and 0.2 (1.0). The annualized mean LOS (in days) for them by corresponding line was 1.6 days (SD=9.8 days), 2.1 days (8.8 days), 3.2 days (8.2 days), 3.7 days (7.1 days), and 3.1 days (6.5 days). The number of patients that experienced all-cause ER visit were 363 (60.7%) for first-line, 93 (51.7%) for second-line, 25 (49.0%) for third-line, 11 (52.4%) for fourth-line, and 5 (50%) for fifth-line therapy, respectively. The annualized mean number of all-cause ER visits for these patients by corresponding line were 0.7 (SD=1.8) for first-line, 0.7 (1.9) for second-line, 1.1 (1.9) for third-line, 0.7 (0.9) for fourth-line, and 0.9 (1.3) for fifth-line therapy, respectively. No significant numbers of FL-related hospitalization and FL-related ER visits were observed.

The highest HCRU categories both for the all-cause and FL-related analyses were for office and other visits (per patient per year). Almost all patients experienced all-cause physician office visits and other outpatient visits in the follow-up period. All-cause office visits ranged from 15.6 in first-line to 20.1 for fifth-line therapy. All-cause other outpatient visits ranged from 28.1 for first-line to 38.1 for fifth-line therapy. As was observed with costs, all-cause other visits were high in third- and fourth-line therapies. These visits were likely associated with chemotherapy administration. FL-related office visits ranged from 3.6 for first-line to 6.7 for fourth-line therapy. FL-related other visits ranged from 4.6 for first-line to 8.5 for fourth-line therapy.

## DISCUSSION

The average age at FL diagnosis reported previously of 60 years is consistent with the current results, which found the mean age at first-line therapy was 60.7 years.^19^,^47^ The vast majority of patients in this analysis had FFS health plans. The mean CCI score of 3.2 indicates that patients in this cohort had a moderate risk of mortality.^48^ The most common comorbidities were other malignancies, which is consistent with what could be expected from a FL patient cohort.^19^,^47^

In terms of treatment distributions from first- to fifth-line therapies, there was a general tendency for subsequent lines of therapy to have shorter durations of treatment. The exception was for second-line therapy, which saw an increase in the mean of treatment duration from 370 days (for first-line therapy) to 392 days. In this instance, the median IQR values were higher than the mean values suggesting that many patients needed urgent treatment. In other lines of therapy (other than second-line therapy), the median values tended to be less than the mean indicating a less urgent need for treatment. Meyer et al. reported similar findings in a study of indolent NHL, including FL, with relapsing courses after initial therapy.^20^ The authors observed that for relapsing patients, the response duration to subsequent lines of therapy shortened with time and patients eventually became more refractory to treatment.

The combination of rituximab and chemotherapy has become the standard of care for FL patients needing first-line or second-line therapy.^22^,^49^,^50^ Not surprisingly, the current study found rituximab monotherapy and combination therapy (R-CHOP, BR, R-CVP, FCR, cyclophosphamide plus rituximab) to be the most prevalent at every line of treatment in this FL patient cohort. Several other recent studies have confirmed the rituximab monotherapy or combination therapy as the most prevalent treatment option.^20^,^51^–^53^ Link et al. found this to be the case for first-line to fifth-line treatments in refractory FL patients from the National LymphoCare Study.^51^ However, a substantial proportion of those patients on rituximab-containing therapy in the current study went on to require multiple subsequent lines of therapy, confirming the substantial health burden on patients with the diagnosis.

FL relapse and progression are associated with poor patient outcomes.^7^ Binkely et al. reported that patients who relapse within 1 to 2 years of treatment have a worse prognosis than those who do not.^7^ For patients who received first-line R-CHOP, FL disease progression within 2 years after diagnosis was associated with poor patient outcomes.^54^ Similarly, Bruna and colleagues found that achieving remission was associated with increased odds of survival.^52^

Results from the current study underscore the significant financial burden of FL. The annualized cost of treatment lines in our study ranged from US$100 000 to US$425 000. Chemotherapy and pharmacy costs made up the highest proportions of these therapy costs. Other studies have highlighted the great economic burden of FL treatment. Morrison et al. conducted a study to evaluate HCRU of newly diagnosed FL patients from 2008 to 2015 from the Optum claims database.^27^ Overall, their mean per-patient, per-month cost during 2 years of follow-up was US$10 460. Consistent with the current study, Morrison et al. reported a reduction in both HCRU and costs from year 1 to year 2. The study also found the largest driver of medical costs was chemoimmunotherapy.^27^ Here, we provide additional data that extend beyond 2 years, showing that costs increased in the third line through fifth line of therapy. The first line of therapy was almost 1 year long. In a study from 2004–2013 with Truven Health MarketScan Commercial and Medicare claims databases using ICD Codes, Meyer et al. found that the mean annual total medical cost for patients with relapsing indolent NHL was US$170 179.^20^ In their study, the cost proportions were: 62% outpatient, 34% inpatient, and 4% outpatient pharmacy.^20^ Maziarz et al. reported high HCRU and total average health care costs of US$455 741 during the first year in a study of DLBCL patients.^55^ These costs had a tendency to decrease in year 2 and year 3 but still remained substantial (US$92 720 and US$72 957, respectively). These results from the published literature and current study demonstrate the high economic burden associated with multiple lines of FL therapy.

## LIMITATIONS

This study presents some limitations that are discussed herein. First, the sample size was relatively small for this study, which may reduce the power of the study. For example, although 598 patients were initiated on FL indicated therapies, only 21 patients received fourth-line therapy and 10 patients received fifth-line therapy. Therefore, estimation of treatment distribution, costs, and HCRU for these lines of therapy may not have been stable. In addition, transplants are costly procedures for FL treatment. Second, the follow-up time was relatively short. FL is typically a slow-growing or indolent form of NHL and is considered a chronic disease. The course of treatment lasts for over 20 years and patients can undergo over 10 lines of therapy. This study only followed patients for 5.7 years and patients only received five lines of therapy. This economic burden of FL was likely underestimated. Third, since data for this study were collected in earlier years, newly approved FL regimens were not included in this study. For instance, only patients who were initiated on a FL indicated regimen between January 1, 2010 through December 31, 2013 were included in this study as index regimens and were followed up for evaluating the burden of illness. Medications approved after 2013 (eg, such as Idelalisib, copanlisib, duvelisib) were not included as index regimen but only as switched regimens. Therefore, treatment profile and economic burden associated with most recent FL indicated regimens may be under-reported in this study. Fourth, the generalizability of the study results is limited because patients excluded from this study may be different from patients who were included. Fifth, due to the sample size and availability of the data, we could only follow-up patients until the fifth line of therapy. Due to this, the FL may have become more severe and the costs would have been higher if given a longer follow-up period. Unfortunately, we did not have the data to evaluate the patients’ severity of FL disease even within the follow-up period. Finally, this study was based on claims data mainly collected from population who had employer sponsored insurance as their primary or as supplementary coverage to their Medicare insurance. Government-sponsored Medicare, Medicaid, and other type of insured or uninsured population were not included this study, and thus caution should be taken when drawing conclusions from this study. More studies are necessary to address these limitations.

## CONCLUSION

The results from this study serves as real-world data and show that primary FL regimens used across treatment lines in this study conform to NCCN guidelines recommendations. In addition, the economic burden for patients with FL is high, and tends to increase with subsequent lines of chemotherapy.

## Supplementary Information



## Figures and Tables

**Table 1:** Patient Characteristics in Baseline Period (N=598)

Characteristics	
**Age (in years), mean (SD)**	60.7 (13.1)
**Age (in years), median (IQR)**	60.0 (19.0)
**Female, n (%)**	300 (50.2)
**Region, n (%)**	
**Northeast**	90 (15.1)
**Midwest**	207 (34.6)
**South**	218 (36.5)
**West**	83 (13.9)
**Health Insurance Plan, n (%)**	
**Fee for Service**	515 (86.1)
**HMO and POS Capitation**	82 (13.7)
**CCI Score, mean (SD)**^a^	3.2 (1.9)
**CCI Score, median (IQR)**	3.0 (2.0)
**CCI by category, n (%)**	
**Myocardial Infarction**	16 (2.7)
**Congestive Heart Failure**	27 (4.5)
**Peripheral Vascular Disease**	35 (5.9)
**Cerebrovascular Disease**	44 (7.4)
**Dementia**	1 (0.2)
**Chronic Pulmonary Disease**	102 (17.1)
**Rheumatic Disease**	24 (4.0)
**Peptic Ulcer Disease**	10 (1.7)
**Mild Liver Disease**	35 (5.9)
**Diabetes without Chronic Complications**	116 (19.4)
**Diabetes with Chronic Complications**	19 (3.2)
**Hemiplegia or Paraplegia**	1 (0.2)
**Renal Disease**	34 (5.7)
**Moderate or Severe Liver Disease**	2 (0.3)
**Metastatic Solid Tumor**	40 (6.7)
**HIV/AIDS**	0 (0)
**Other Malignancy**	571 (95.5)

Abbreviations: CCI, Charlson Comorbidity Index; HIV/AIDS, human immunodeficiency virus infection and acquired immune deficiency syndrome; IQR, interquartile range; POS, point of service; SD, standard deviation.

**Table 2:** Treatment Duration by Line of Therapy (in days)

Line of Therapy	Mean (SD)	Median (IQR)
First-line therapy (n=598)	370 (467)	141 (602)
Second-line therapy (n=261)	392 (435)	259 (541)
Third-line therapy (n=72)	162 (233)	85 (123)
Fourth-line therapy (n=24)	148 (227)	42 (84)
Fifth-line therapy (n=11)	88 (151)	21 (133)

Abbreviations: IQR, interquartile range; SD, standard deviation.

Required follow-up time was at least 4 years, follow-up time is the time from index date to end of continuous enrollment or diagnosis of diffuse large B-cell lymphoma.

**Table 3:** Regimen Distribution by Line of Treatment

	First-line Therapy (N=598)	Second-line Therapy (N=180)	Third-line Therapy (N=51)	Fourth-line Therapy (N=21)	Fifth-line Therapy (N=10)
**Treatment Agent, n (%)**					
Rituximab	201 (33.6)	78 (43.3)	16 (31.4)	6 (28.6)	2 (20.0)
R-CHOP	143 (24.0)	11 (6.1)	4 (7.8)	2 (9.5)	
BR	143 (24.0)	41 (22.8)	9 (17.8)	1 (4.8)	2 (20.0)
R-CVP	71 (11.9)	12 (6.7)	2 (3.9)	1 (4.8)	
FCR	10 (1.7)	2 (1.1)	1 (2.0)	1 (4.8)	
Cyclophosphamide plus Rituximab	6 (1.0)	13 (7.2)	6 (11.8)		
Fludarabine plus Rituximab	5 (0.8)	1 (0.6)			
Chlorambucil	3 (0.5)	1 (0.6)	1(2.0)		
Lenalidomide	3 (0.5)	2 (1.1)		2 (9.5)	3 (30.0)
Cyclophosphamide	1 (0.2)	2 (1.1)	2 (3.9)		
Fludarabine	1 (0.2)	4 (2.2)		1(4.8)	
Lenalidomide plus Rituximab	1 (0.2)		1 (2.0)	3(14.3)	1 (10.0)
Rituximab plus Transplant	1 (0.2)	5 (2.8)			
Other^a^	9 (1.5)	10 (5.6)	9 (17.6)	4 (19.1)	2(20.0)
Total	598 (100)	180 (100)	51 (100)	21 (100)	10 (100)

Abbreviations: %, percentage; BR, bendamustine plus rituximab; FCR, fludarabine, cyclophosphamide and rituximab; R-CHEOP, rituximab (R), cyclophosphamide (C), hydroxydaunorubicin (H), oncovin (O), etoposide (E), prednisone (P); R-CHOP, rituximab (R), cyclophosphamide (C), doxorubicin hydrochloride (hydroxydaunomycin) (H), vincristine sulfate (oncovin) (O), prednisone (P); R-CVP, rituximab to cyclophosphamide, vincristine, and prednisone, transplants.

a Other refers to combinations of other therapies with fewer than three patients or not clinically indicated in FL (ie, Bendamustine, Ofatumumab, Chlorambucil, Rituximab, Fludarabine, Mitoxantrone, Lenalidomide, Transplant, Ibritumomab Tiuxetan, Idelalisib, Ibritumomab, Obinutuzumab, Chlorambucil, Vincristine).

**Table 4:** Annual Health Care Costs for Patients with FL in the Follow-up Period and Lines of Therapy^a^,^b^

	All Patients at Follow-Up^c^ (N=598)	First-Line Therapy (N=598)	Second-Line Therapy (n=180)	Third-Line Therapy (n=51)	Fourth-Line Therapy (n=21)	Fifth-Line Therapy (n=11)
**Total Health Care Costs, Mean (SD)**^d^	56 831 (55 133)	97 141 (144 730)	125 586 (278 654)	239 216 (548 666)	370 597 (718 510)	424 758 (715 028)
**Medical Costs, Mean (SD)**^e^	50 925 (49 851)	91 567 (143 091)	116 566 (277 073)	222 686 (551 657)	275 817 (671 979)	351 892 (724 222)
**Inpatient Visits**	5416 (13 821)	8132 (57 990)	13 347 (65 897)	11 357 (27 185)	15 331 (31 893)	17 827 (36 579)
**Emergency Care**	1108 (3233)	1670 (10 503)	1364 (4842)	1359 (2798)	1159 (2174)	2253 (4109)
**Office Visit Costs**	1627 (1180)	2125 (2483)	2005 (1692)	2763 (4031)	1950 (1300)	2537 (2311)
**Outpatient Costs, Mean (SD)**						
**Chemotherapy Drugs**^f^	26 284 (35 463)	55 298 (106 995)	68 377 (221 547)	92 648 (147 050)	163 864 (393 490)	112 146 (169 753)
**Diagnosis**^i^	4907 (6976)	7119 (13 859)	7825 (16 858)	44 907 (277 700)	10 591 (11 851)	8220 (6878)
**Medical Services**^k^	4627 (5490)	8313 (17 811)	16 095 (103 286)	58 595 (337 313)	77 418 (281 538)	205 462 (626 195)
**Surgery**	2363 (3533)	3082 (12 118)	3131 (5929)	1849 (3145)	1800 (2468)	2787 (2819)
**Other**^i^	4596 (13 880)	5832 (16 993)	4425 (13 764)	9209 (42 679)	3705 (7492)	659 (1571)
**Total Pharmacy Costs, Mean (SD)**^j^	5905 (15 937)	5573 (17 068)	9020 (36 518)	16 530 (41 144)	94 780 (193 984)	72 865 (128 966)
**Total FL-Related Health Care Costs, Mean (SD)**	13 821 (19 173)	29 557 (83 633)	29 460 (109 186)	103 387 (406 719)	309 103 (722 131)	85 748 (131 478)
**FL-Related Medical Costs, Mean (SD)**^k^	4496 (9918)	8627 (23 159)	6172 (24 872)	57 670 (327 059)	27 511 (52 680)	1850 (5215)
**FL-Related Inpatient Visits**	605 (4154)	924 (8826)	1871 (22 591)	5053 (22 098)	2979 (10 936)	447 (1412)
**FL-Related Emergency Care Visits**	32 (222)	32 (265)	14 (126)	117 (519)	0 (0)	0 (0)
**FL-Related Outpatient Visits**	3859 (8826)	7671 (21 416)	4288 (10 667)	52 501 (327 113)	24 532 (51 108)	1403 (3808)
**FL-Related Office Visits**	285 (719)	499 (1472)	332 (710)	995 (3826)	424 (918)	270 (741)
**FL-Related Other Visits**	3574 (8547)	7172 (20 598)	3955 (10 390)	51 506 (327 038)	24 108 (51 118)	1133 (3071)
**Costs Of FL-Related Transplant Cell in Any Medical Setting**	12 (194)	3 (64)	3 (40)	109 (776)	50 (230)	0 (0)
**FL-Related Total Pharmacy Costs, Mean (SD)**^l^	9325 (14 094)	20 930 (67 618)	23 288 (105 083)	45 717 (112 893)	281 593 (687 685)	83 898 (130 605)
**Costs Of FL-Related Drugs in Any Medical Setting**^m^	8534 (12 637)	19 990 (66 916)	19 521 (100 468)	36 131 (109 983)	195 616 (648 897)	14 720 (46 550)
**Cost Of FL-Related Oral Drugs**	791 (6490)	941 (11 381)	3767 (32 996)	9586 (36 494)	85 77 (197 500)	69 177 (130 972)

Note: Annualized costs were computed as: (sum of all costs of interest in the time frame of interest)/total number of member months of the time frame ×12. For example, annualized mean medical costs in follow-up period were computed as (1) the sum of all medical costs in the follow-up period, (2) the sum of the member month (if a patient was followed up for 5 years, he/she contributed 12×5=60 member months), (3) the divided sum of all medical costs by sum of member months and the number of per-member, per-month medical costs, and (4) then multiply this number by 12 months.

a Health care costs were measured from a payer’s perspective and are reported in 2018 US dollars.

b Health care costs were defined as the total costs occurring during the studied line of therapy reported on a yearly basis to account for different durations of line of therapy.

c Costs in specific category in the follow-up period included the costs incurred in the entire follow-up period, regardless of the line of treatment.

d Total health care costs are defined as all direct medical costs related to treatment in inpatient, outpatient, and physician office as well as pharmacy costs.

e Medical costs are defined as direct medical costs related to treatment in inpatient, outpatient, and physician office.

F Chemotherapy included chemotherapy drugs and temporary drug codes.

g Diagnosis included laboratory, pathology, and radiology services.

h Medical services include medicine services, devices, durable and rehab services, enteral therapy, anesthesia, and surgery.

i Other includes miscellaneous and transportation services.

j Pharmacy costs were costs derived from all medications used in follow-up period.

k FL-related medical costs are defined as direct medical costs related to treatment of FL diseases in inpatient, outpatient, and physician office.

l FL-related total pharmacy costs were derived from pharmacy claims based on the list of systemic anti-cancer therapies (Supplementary Material).

m Costs of FL-related drugs in any medical setting are were derived from medical claims based on the list of systemic anti-cancer therapies (Supplementary Material).

**Table 5:** Frequency of Annual Total Health Care Resource and Utilization for Patients with FL in the Follow-up Period and Lines of Therapy^b^

	Follow-Up Period (N=598)	First-Line Therapy (N=598)	Second-Line Therapy (N=180)	Third-Line Therapy (N=51)	Fourth-Line Therapy (N=21)	Fifth-Line Therapy (N=10)
**All-Cause Hospitalization, n (%)**	281 (47.0)	228 (38.1)	56 (31.1)	19 (37.3)	10 (47.6)	5 (50.0)
**All-Cause Hospitalization, Mean (SD)**	0.2 (0.3)	0.2 (1.0)	0.5 (2.6)	0.5 (1.1)	0.7 (1.4)	0.2 (0.3)
**All-Cause LOS (Inpatient), Mean (SD)**^a^	1.1 (2.1)	1.6 (9.8)	2.1 (8.8)	3.2 (8.2)	3.7 (7.1)	3.1 (6.5)
**All-Cause ER Visits, n (%)**	440 (74.0)	363 (60.7)	93 (51.7)	25 (49.0)	11 (52.4)	5 (50.0)
**All-Cause ER Visits, Mean (SD)**	0.6 (1.0)	0.7 (1.8)	0.7 (1.9)	1.1 (1.9)	0.7 (0.9)	0.9 (1.3)
**All-Cause Office Visits, n (%)**	597 (99.8)	593 (99.2)	176 (97.8)	48 (94.1)	19 (90.5)	8 (80.0)
**All-Cause Office Visits, Mean (SD)**	12.6 (6.9)	15.6 (13.6)	15.2 (11.2)	17.6 (12.0)	19.9 (27.7)	20.1 (19.2)
**All-Cause Other Outpatient Visits, n (%)**	598 (100)	598 (100)	179 (99.4)	51 (100)	20 (95)	8 (80)
**All-Cause Other Outpatient Visits, Mean (SD)**	21.2 (14.1)	28.1 (28.1)	30.4 (27.7)	34.0 (20.5)	37.0 (25.5)	36.1 (29.0)
**FL-Related Hospitalization, n (%)**	4 (0.7)	2 (0.3)	0 (0)	2 (4)	0 (0)	0 (0)
**FL-Related Hospitalization, Mean (SD)**	0.001 (0.02)	0.001 (0.01)	0.0 (0.0)	0.0 (0.1)	0.0 (0.0)	0.0 (0.0)
**FL-Related LOS (Inpatient), Mean (SD)**^a^	0.03 (0.4)	0.01 (0.22)	0.0 (0.0)	0.4 (2.2)	0.0 (0.0)	0.0 (0.0)
**FL-Related ER Visits, n (%)**	46 (7.7)	32 (5.4)	9 (5.0)	4 (7.8)	2 (9.5)	(0.0)
**FL-Related ER Visits, Mean (SD)**	0.02 (0.1)	0.02 (0.2)	0.02 (0.1)	0.25 (1.1)	0.06 (0.3)	0.0 (0.0)
**FL-Related Office Visits, n (%)**	446 (74.6)	419 (70.1)	110 (61.1)	28 (54.9)	12 (57.1)	3 (30.0)
**FL-Related Office Visits, mean (SD)**	1.9 (2.1)	3.6 (9.1)	2.4 (4.6)	4.7 (9.5)	6.7 (19.8)	1.3 (3.6)
**FL-Related Outpatient Visits, n (%)**	431 (72.1)	410 (68.6)	104 (57.8)	20 (39.2)	12 (57.1)	3 (30.0)
**FL-Related Outpatient Visits, Mean (SD)**	2.1 (2.7)	4.6 (11.3)	2.8 (5.7)	4.1 (13.3)	8.5 (20.0)	2.6 (7.5)

Abbreviations: ER, emergency room; FL, follicular lymphoma; LOS, length of stay; SD, standard deviation.

a Frequency or LOS is measured on yearly basis (frequency [LOS]) in specified period/365) “based on all patients” means the patients without specified resource utilization with be assigned 0 and “based on patient with ≥” means only patients with at least one visit are counted when computing the mean, median, etc. Patients with 0 utilization of health care resource utilization were included in the analysis.

b Annualized HCRU was computed as: (the sum of all events of interest in the time frame of interest)/the total number of member months in the time frame ×12. For example, the mean number of all-cause of hospitalizations in the follow-up period was computed as (1) the sum of all hospitalization events, (2) the sum of member months (if a patient was followed up for 5 years, he/she contributed 12×5=60 member months), (3) divided by the sum of all hospitalization events by the sum of member months to get the number of per-member, per-month hospitalization rate, (4) multiplied by this number by 12 months.
