# Supplementary material for: Treatment Patterns and Health Care Costs in Commercially Insured Patients with Follicular Lymphoma
Source: J Health Econ Outcomes Res. 2020 Sep 4;7(2):148–57. doi: 10.36469/jheor.2020.16784 (PMC7539759; doi:10.36469/jheor.2020.16784)
Supplement: Supplementary file 1 [file jheor-7-2-16784-s01.pdf]

Journal of Health Economics and Outcomes Research

Supplementary Online Material

Fowler NH, Chen G, Lim S, Manson S, Ma Q, Li FY. Treatment patterns and health care costs in commercially insured patients with follicular lymphoma. *JHEOR*. 2020;7(2):148-157.
doi:10.36469/jheor.2020.16784

Table S1. Recommended Treatment Regimens

This supplementary material has been provided by the authors to give readers additional information about their work.

This is an open-access article distributed under the terms of the Creative Commons Attribution 4.0 International License (CCBY-4.0). View this license's legal deed at http://creativecommons.org/licenses/by/4.0 and legal code at http://creativecommons.org/licenses/by/4.0/legalcode for more information.

Table S1. Recommended Treatment Regimens

<table>
<tr><th>List of NCCN Recommended Agents for Patients with FL</th><th>Drug Class</th><th>First-Line Regimens</th></tr>
<tr><td>Duvelisib</td><td>Kinase inhibitor</td><td rowspan="8">Fit Adults
Preferred regimens
• Bendamustine and (obinutuzumab or rituximab)
• CHOP and (obinutuzumab or rituximab)
• CVP and (obinutuzumab or rituximab)
• Lenalidomide and rituximab
Other regimens
• Rituximab</td></tr>
<tr><td>Rituximab</td><td>Monoclonal antibody</td></tr>
<tr><td>Obinutuzumab</td><td>Cytolytic antibody</td></tr>
<tr><td>Bendamustine</td><td>Chemotherapy</td></tr>
<tr><td>Cyclophosphamide</td><td>Chemotherapy</td></tr>
<tr><td>Doxorubicin</td><td>Chemotherapy</td></tr>
<tr><td>Vincristine</td><td>Chemotherapy</td></tr>
<tr><td>Prednisone</td><td>Corticosteroid</td></tr>
<tr><td>Lenalidomide</td><td>Immunomodulator</td><td rowspan="8">Frail Adults
Preferred regimens
• Rituximab
Other regimens
• Chlorambucil and rituximab
• Cyclophosphamide and rituximab
• Chlorambucil
• Cyclophosphamide
• Ibritumomab tiuxetan</td></tr>
<tr><td>Fludarabine</td><td>Chemotherapy</td></tr>
<tr><td>Mitoxantrone</td><td>Chemotherapy (antitumor antibiotic)</td></tr>
<tr><td>Ofatumumab</td><td>Antibody</td></tr>
<tr><td>Ibritumomab tiuxetan</td><td>Radioimmunotherapy</td></tr>
<tr><td>Chlorambucil</td><td>Chemotherapy</td></tr>
<tr><td>Idelalisib</td><td>Kinase inhibitor</td></tr>
<tr><td>Copanlisib</td><td>Kinase inhibitor</td></tr>
</table>

Abbreviations: NCCN, National Comprehensive Cancer Network.
